# Lipid peroxidation in diamond supported bilayers†

**DOI:** 10.1039/d3nr01167d

**Published:** 2023-04-11

**Authors:** A. R. Ortiz Moreno, R. Li, K. Wu, R. Schirhagl

**Affiliations:** a Groningen University, University Medical Center Groningen Antonius Deusinglaan 1 9713 AW Groningen the Netherlands romana.schirhagl@gmail.com

## Abstract

Lipid peroxidation is a process that occurs in cells when they are exposed to oxidative stress. During the process reactive oxygen species attack lipids within the lipid bilayers of cells. Since the products of lipid peroxidation are toxic and carcinogenic, it is important to understand where and how it occurs with nanoscale resolution. The radical intermediates of this process are particularly interesting since they are causing chain reactions damaging large parts of the lipid membranes in cells. However, they are also difficult to measure for the state of the art because they are short lived and reactive. Here, we study the lipid peroxidation of three artificial lipid bilayers on a diamonds substrate that can be used to study lipid peroxidation. In particular, we present a diamond quantum sensing method called *T*_1_-relaxometry that allows for *in situ* measurements and imaging of radical intermediates of lipid peroxidation in these membranes.

## Abstract 2

Here we investigate lipid peroxidation at the nanoscale using diamond based relaxometry in a lipid bilayer.

## Graphical abstract

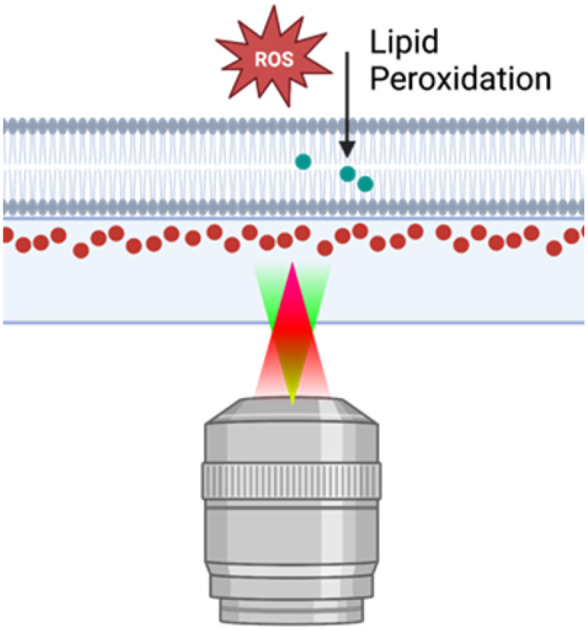

## 1. Introduction

Artificial membranes provide a useful model for biological structures and their applications, since they act as a simplified version of the cell that can change their properties through chemical or physical processing.^1,2–4^ One of the phenomena that can be studied using these membranes is lipid peroxidation, a reaction in which oxidants attack lipids with a carbon–carbon double bond.^5^ In cells, it is one of the main molecular mechanisms involved in oxidative damage,^6^ mainly due to the presence of free radicals that change the physical properties of the membrane and the fact that these radicals cause chain reactions which can damage large parts of the membranes.^7^ While lipid peroxidation has been tested through the quantification of the end-products,^8^ there is not much research on online detection of it.

**Fig. 1:**
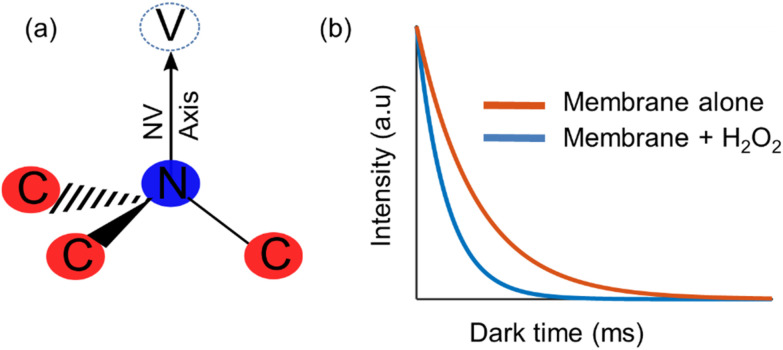
Diamond quantum relaxometry. (a) Physical structure of the nitrogen-vacancy (NV) center in diamond (b) *T*_1_ relaxometry curve with and without the presence of paramagnetic species (in this case radical intermediates from lipid peroxidation).

On the other hand, diamond quantum sensing has proven to be an effective and sensitive tool for nanoscale metrology of several quantities in biochemical media, such as intra-cellular temperature,^9^ spin labels in cell membranes,^10^ magnetic imaging of living cells,^11^ among others. The study of these has been enabled through the nitrogen-vacancy (NV) centre (Fig. 1a), which is a colour centre that acts as an isolated atom inside the diamond matrix, and can emit fluorescence that depends on their quantum state. All of these factors combined make diamond an ideal sensor for non-invasive, nanoscale, room-temperature biochemical phenomena.^12^

In order to use the NV centre as a sensor for the detection of a chemical reaction, it is required to implement a protocol that is sensitive to the magnetic field fluctuations produced during a chemical reaction, which have a noise spectrum of a few Gigahertz.^10^ One such protocol is measuring the relaxation time of the NV centre ensemble, which corresponds to the time in which a polarized ensemble (in the *m*_s_ = 0) returns to their original thermal distribution. This protocol is usually referred to as *T*_1_ relaxometry, and it is of special interest in biological research due to the fact that is microwave free. In this protocol, the NV centre is polarized using a near resonant excitation (532 nm wavelength), and then is allowed to evolve freely for a certain amount of time (dark time) without any driving.^13,14^ After that time has elapsed, the NV centre is excited once again in order to optically read the NV centre states, the higher the intensity the more defects in the *m*_s_ = 0 state (and *vice versa*). This behaviour can be characterized through a relaxometry curve (Fig. 1B), alongside its characteristic *T*_1_ time that decreases in the presence of more free radicals.^15,16,17^

**Fig. 2:**
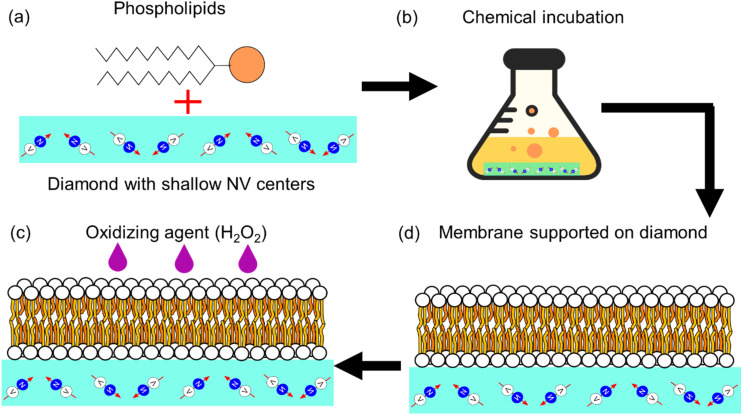
Overview of the planar lipid bilayer preparation and oxidation experiment. (a) Lipid structure and diamond sample with NV centres near the surface (b) Chemical incubation step. The lipid bilayer is grown right on top of the diamond sensor (c) Final structure. The lipid bilayer is supported in the diamond sensor (d) An oxidizing agent is added in order to initiate the lipid peroxidation process.

Although diamond detection of nuclear magnetic resonances, a different diamond based sensing scheme, has been previously used for measuring phase transitions in lipid bilayers in,^18^ it has not been used to probe the presence of free radicals in such systems. In this study, we present a new approach that uses diamond quantum relaxometry (or *T*_1_ relaxometry) to measure lipid peroxidation *via* the sensing of free radicals generated in an artificial lipid bilayer grown directly on the diamond. A schematic representation of this study is shown in Fig. 2.

## 2. Materials & methods

### 2.1. Diamond sample and preparation

A 2 × 2 × 1 mm^3^ electronic grade diamond sample from Element Six was acquired for these experiments. In order for the diamond to sense the presence paramagnetic species on the surface *via* quantum relaxometry, the defects must be close to the surface (up to some tens of nm^19,20^). To generate NV centers close to the surface, the diamond sample was implanted with diatomic nitrogen (N_2_) with an implantation energy of 5 keV and a dose of 10^9^ 1 cm^−2^. After implantation, the diamond was annealed at 800 °C for 4 hours allowing the vacancies to diffuse across the diamond. This process creates a dense layer of nitrogen vacancy centres approximately 12 nm below the diamond surface.

Diamond plates were cleaned with a mixture of sulfuric acid and nitric acid (1 : 3 H_2_SO_4_/HNO_3_) at 140 °C for 4 hours. Then plates were rinsed with ultrapure water to get rid of residual acid of plate surface.

### 2.2. Preparation of the lipid bilayer and H_2_O_2_

**Fig. 3:**
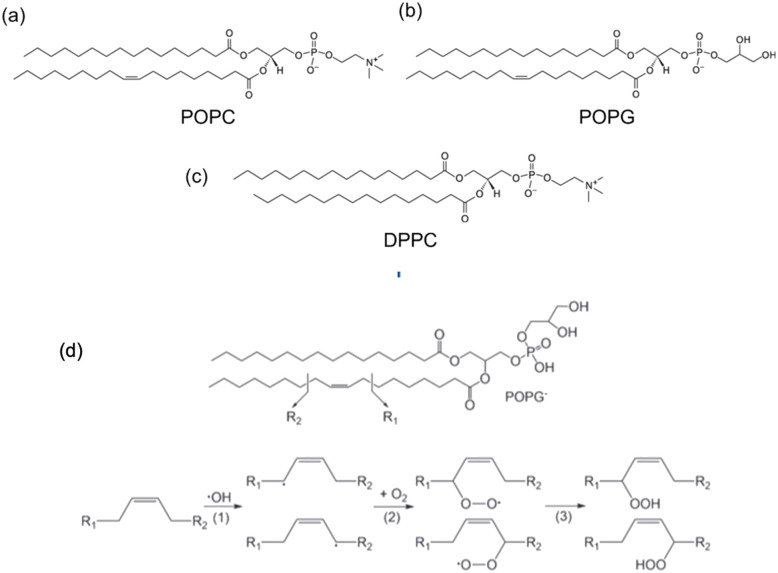
Phospholipids used to detect lipid peroxidation in artificial membranes. (a) POPC (b) POPG and (c) DPPC. The lipids on the top have a double bond in the alkyl chain that allows for lipid peroxidation. (d) Mechanism of lipid peroxidation for POPG as an example.

1,2-Dipalmitoyl-*sn-glycero*-3-phosphocholine (DPPC), 2-oleoyl-1-palmitoyl-*sn-glycero*-3 phosphocholine (POPC), 1-palmitoyl-2-oleoyl-*sn-glycero*-3-[phospho-*rac*-(1-glycerol)] (POPG) were purchased from Sigma (see Fig. 3). DPPC was dissolved in chloroform in a round-bottom flask and we removed the solvent in a rotary evaporator. As a result, we obtained a thin film of lipid covering the flask. Then Milli-Q water was added to this lipid thin film and we sonicated it for 30 min by water bath sonication to detach it from the bottom surface of the flask. DPPC vesicles were formed by extruding the suspension 20 times with a vesicles extruder through a 400 nm pore-size polycarbonate membrane. The obtained liposomes with uniform particle size were diluted to 1 mg mL^−1^, then 20 μL of suspension were dropped onto the diamond surface and incubated for 2 h at 60 °C in an oven. After incubation, the plate was washed several times with Milli-Q water to remove unadhered free lipids. POPC and POPG bilayers were prepared using the same method. In order to assess the particle size and uniformity, dynamic light scattering measurements were performed (see ESI Fig. S4†).

The oxidizing agent was prepared by mixing a 30% solution of hydrogen peroxide with water, in order to achieve the required concentration.

### 2.3. *T*_1_ relaxometry

The relaxometry data was acquired using a homebuilt, wide-field microscope designed for relaxometry in diamond NV-centres. Optical excitation from a 520 nm diode laser (Dilas) is sent through a fibre optic with an integrated collimator (Thorlabs F230SMA). When the excitation reaches the microscope, the beam is focused to the sample using a wide-field lens (*f* = 200 mm) to reach the diamond sample from the top. The NV fluorescence is collected using a Mitutoyo 50×, 0.65 NA microscope objective, a 175 mm focal field for further focusing and a 650 nm long-pass filter in front of the SCMOS camera (Andor zyla).

The pulsing sequence for the relaxometry protocol was performed through the internal switching of the laser diode.

The instrument is controlled using a home-made LabVIEW program to automatically acquire the *T*_1_-relaxation curves and images.

The relaxometry protocol used with the widefield setup is very similar to the typical sequence used in an NV center confocal setup.^21^ It consists of a polarization pulse that pumps all the NV centers to the *m*_s_ = 0 state, followed by a readout pulse that allows for the detector to read the current state population of the NV centers. However, the counts we detect are not completely zero when the light source is off. As a result, for longer dark times, these dark counts would give a brighter image with a higher exposure time, and therefore assigning higher fluorescence to higher dark times.

To compensate for this artefact, the sequence is applied *N* times per each sampled dark time, then the same sequence is applied again but with a much lower laser power in order to remove the background from the images. Fig. 2b shows this adapted protocol, where the sequence first (data) is labelled as “HIGH” and the reference sequence (background) as “LOW”.

After the fluorescence data has been collected, we use a monoexponential fit^22^ to obtain the *T*_1_ relaxation time of the form1
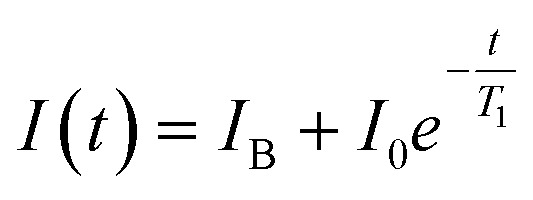


### 2.4. Image and data analysis

The relaxometry data was analysed using a customized series of scripts in MATLAB. These scripts turn a set of images that are encoded using the dark time to a new cell from the same size that consists of ‘pixels’ of relaxometry data, instead of intensity data. For measuring lipid peroxidation, the image is binned altogether in order to obtain a higher signal to noise ratio. On the other hand, for the relaxometry imaging of the membranes, the image is binned into 4 × 4 pixels instead.

### 2.5. Confocal imaging

In order to asses surface coverage, fluorescent lipid bilayers of DPPC, POPC, POPG were prepared by mixing 1 wt% Rhodamine-PE with phospholipids in chloroform in a round-bottom flask followed by solvent evaporation by rotary evaporator. The rest of the steps were the same as with preparing non-fluorescent lipid bilayers. Images were collected by a Zeiss LSM780 confocal microscope at 561/580 nm of excitation/emission wavelength.

### 2.6. AFM imaging

20 μL of DPPC, POPC, POPG suspension prepared above were dropped onto the smooth surface of diamond plates, and we incubated them to promote lipid bilayer formation for 2 h in a 60 °C oven. Morphology images were obtained by a BioScope Catalyst AFM system (Bruker Nano, Santa Barbara, CA) with tapping mode in air (DNP-10 tip). Then lipid bilayers were treated with 2% H_2_O_2_ solution. Bilayer images were collected again by AFM. All images were analysed using the Nanoscope Analysis 1.8 software, and their roughness were calculated using Root mean square roughness qualification (*R*_q_).

### 2.7. XPS Spectroscopy

The sample membranes described above were prepared on a gold coated glass slide in order to check the surface chemistry of the samples. The XPS machine (S-Probe, Surface Science Instruments, Mountain View, CA, United States) operates in the pre-vacuum range (10^−9^ Pa). X-rays (10 kV, 22 mA) at a spot size of 1200 × 500 μm were produced using an aluminium anode. Scans of the overall spectrum in the binding energy range of 0–1100 eV were made at low resolution (pass energy 150 eV).

### 2.8. Statistics

GraphPad Prism 8.0 was used to perform statistical analysis of *T*_1_ data with an unpaired *t*-test of the Mann–Whitney test between two groups. *T*_1_ data were presented as the mean ± standard deviation. The statistical results are shown as: *P ≤ 0.05, **p ≤ 0.01, ***p ≤ 0.001, **** *p* ≤ 0.0001 and ns indicates non-significance.

## 3. Results and discussion

### 3.1. Effect of the oxidizing agent

To measure lipid peroxidation, first it is necessary to control for the other sources of magnetic noise. It has already been shown in the literature that H_2_O_2_ can decrease the relaxation time when producing *OH radicals *via* photolysis Here we investigate lipid peroxidation at the nanoscale using diamond based relaxometry in a lipid bilayer.^23,24^ To probe this, we measure the relaxation time of the bulk diamond as a function of the hydrogen peroxide concentration (Fig. 5), showing that the only significant effect occurs at 15% concentration weight/volume.

**Fig. 4:**
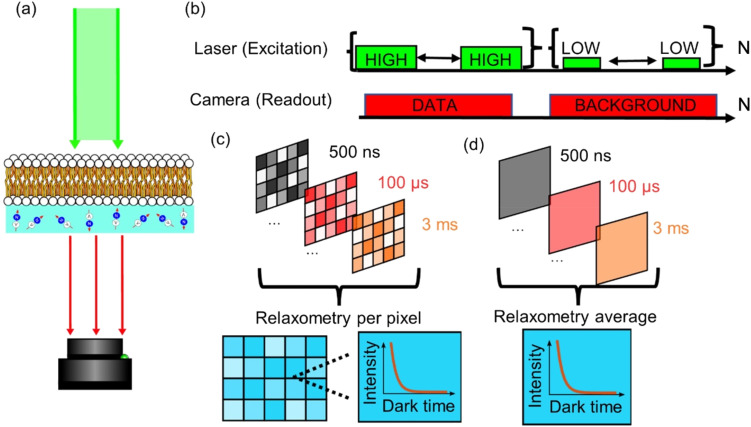
Experimental procedure. (a) Optical readout of the NV center and membrane system (simplified). (b) Pulsed sequence used in order to retrieve the *T*_1_ relaxometry information. (c) Grouping of the images for the relaxometry imaging procedure, in which every pixel contains a relaxometry curve. (d) Grouping of the images for the lipid peroxidation detection. In this case, all the pixels are added together to retrieve a single relaxometry curve.

**Fig. 5:**
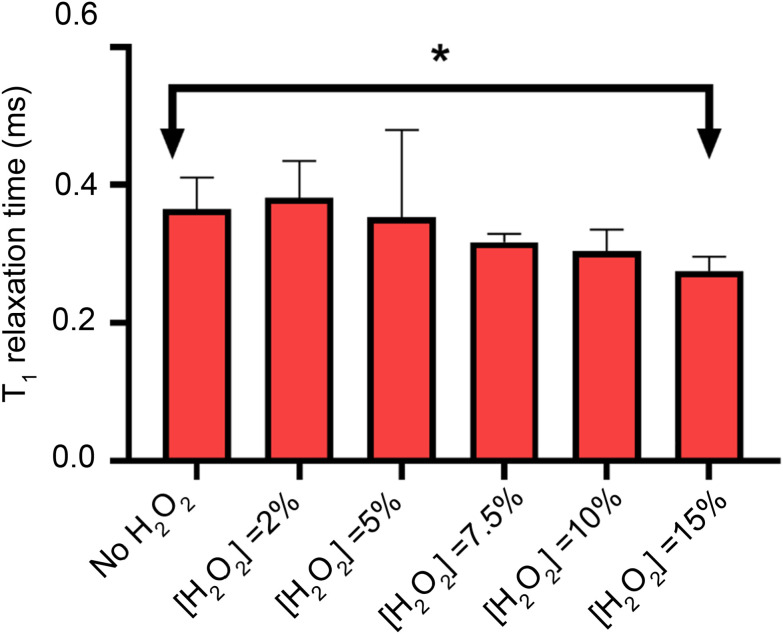
Effect of the hydrogen peroxide in the longitudinal relaxation time as a function of the oxidizing agent concentration. Each experiment shows the *T*_1_ relaxation time at different H_2_O_2_ concentrations, including without H_2_O_2_ (first experiment). Statistical significance (*N* = 6) was assessed with an unpaired *t*-test, showing statistical significant differences only at 15% concentration v/v.

After our control experiment has been performed, we proceed to oxidize the lipid bilayers with 2% H_2_O_2_.

### 3.2. Lipid peroxidation analysis

**Fig. 6:**
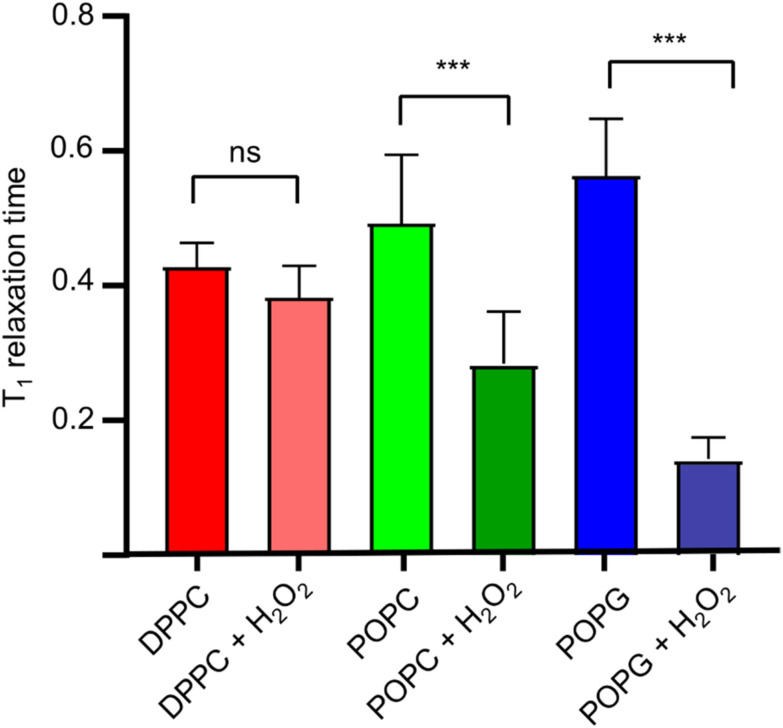
Relaxation times for the diamond and membrane systems, with (immediately after measuring control) and without oxidizing agent (control). Statistical significance (*N* = 6) was assessed with an unpaired *t*-test, having statistical significance only for the POPC and the POPG pairs.

Once we estimated the concentration at which the photodissociation of H_2_O_2_ is negligible for the diamond (2%), we added H_2_O_2_ directly to the lipid bilayer grown on top of the bulk diamond, and then measured the *T*_1_ relaxation time. In Fig. 6, the effect of the oxidizing agent is quantified by averaging the signal from a widefield image in a single value (see Fig. 4d).

The membranes themselves do not contain any paramagnetic components. Thus, as expected, we do not see any significant differences between the bare diamond and after addition in the membranes itself. It is known from literature that lipid peroxidation occurs *via* double bonds within the lipids. Taking this into account we expect that DPPC cannot peroxidise since it does not have a double bond. This is in line with our finding that the *T*_1_ from DPPC is not different before and after addition of 2% H_2_O_2_. POPC and POPG both have one double bond. For both components we see a clear significant decrease in *T*_1_ after the lipids have been peroxidised with H_2_O_2_. We further observed that POPC showed a larger drop in *T*_1_ than POPG. This can have several reasons: first it could be that the surface coverage is different. While we used the same amount of lipid for both lipids, the double bond in POPC could be more reactive than in POPG. It was confirmed later with fluorescence microscopy that POPC covers more of the surface area than POPG. Additionally, there might be differences in in how densely the membrane is packed. This is in line with a finding from Zhang *et al*.^24^ who reported that POPG is packed 20% more densely than POPC. In addition, it has been demonstrated that POPC forms slightly thinner layers with greater mobility.^25^ The result of both phenomena is that lipid peroxidation is sterically hindered and occurs to a lesser extent. It is also worth noting, that this method is based on widefield microscopy only and thus the instrumentation is relatively simple. The data acquisition only took 10–15 minutes.

### 3.3. Relaxometry imaging of the membranes

**Fig. 7:**
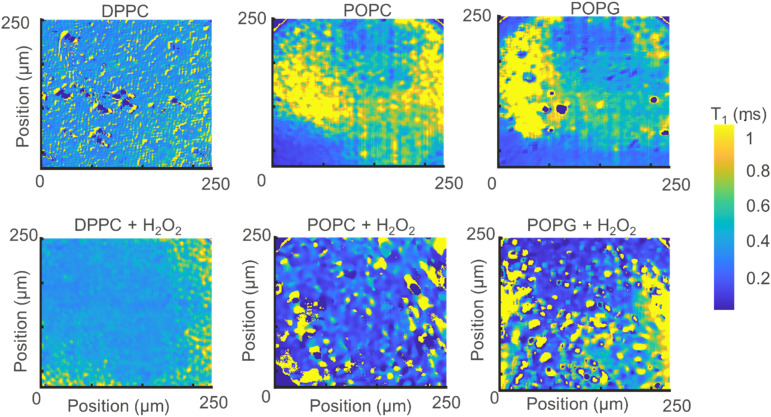
Relaxometry images of the different membranes (up) and the same membranes after exposure to H_2_O_2_ (bottom).

Instead of adding all the pixels from the relaxometry signals provided by the widefield microscope, it is possible instead to analyse them pixel-by-pixel. In Fig. 7, we present an image that, instead of photoluminescence signals, shows the *T*_1_ value (in milliseconds) per binned (4 × 4) pixels.

From Fig. 7 we can make several interesting observations. First, the images without lipid peroxidation are relatively featureless. This is expected since *T*_1_ does not differ if there is a membrane present or not. The lipid membrane itself does not contain any paramagnetic compounds and is thus invisible for this method. The same is true for the sample where DPPC was exposed to H_2_O_2_. This molecule does not contain a double bond in the alkyl chain and thus cannot undergo lipid peroxidation. Another interesting observation here is that we do not see a difference between the bare surface and the DPPC coated surface. These two surfaces have a very different charge environment. If there was an influence of charge state conversion of the NV center on *T*_1_, we would see a shift in *T*_1_ between these two samples. Such effects by charge state conversion have been found for high laser powers or single or very low amounts of NV centers.^26,27^ They do not play a role here as well as in other cases where there are large ensembles and low laser powers (in the μW range).^15,16^

As a result, areas where lipids are present are also unaffected. The situation is different for the other two lipids. These lipids contain a double bond in the alkyl chain which is converted to a radical by H_2_O_2_ by lipid peroxidation resulting in a drop in *T*_1_. As a result, clear patterns appear and the *T*_1_ is shortened.

### 3.4. Confocal images of the stained lipid

**Fig. 8:**
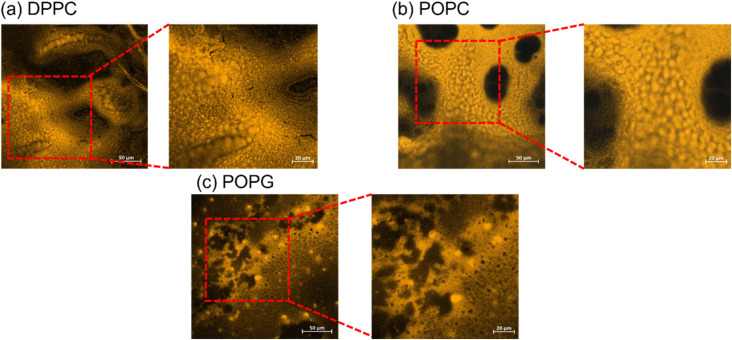
Confocal imaging of the stained lipid bilayers. The bigger images have a size of 300 × 300 μm, while the zoom-in have a size of 150 × 150 μm.

The stained lipid bilayers shown in Fig. 8 show a similar behaviour to the ones obtained in the previous section using the relaxometry imaging technique. We can see from these images that the films are not uniform across the surfaces. Except for DPPC where we do not see any features in the *T*_1_ maps, the images show similar features as the ones recovered from the relaxometry images shown in the previous section. This confirms, that *T*_1_ is indeed sensitive to peroxidation products of the lipids. What we can further conclude from a quantitative analysis of confocal images is a difference in surface coverage (see ESI Fig. S5†).

### 3.5. AFM imaging

**Fig. 9:**
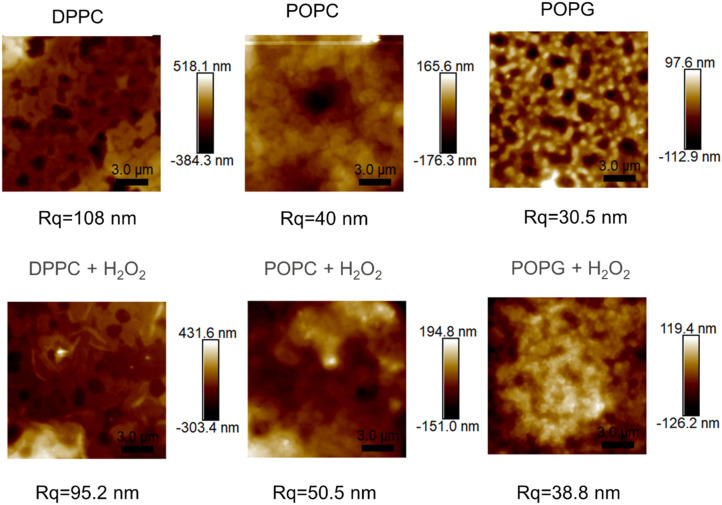
AFM images of the of the different membranes (up) and the same membranes after been exposed to H_2_O_2_ (bottom), alongside their roughness.

**Fig. 10:**
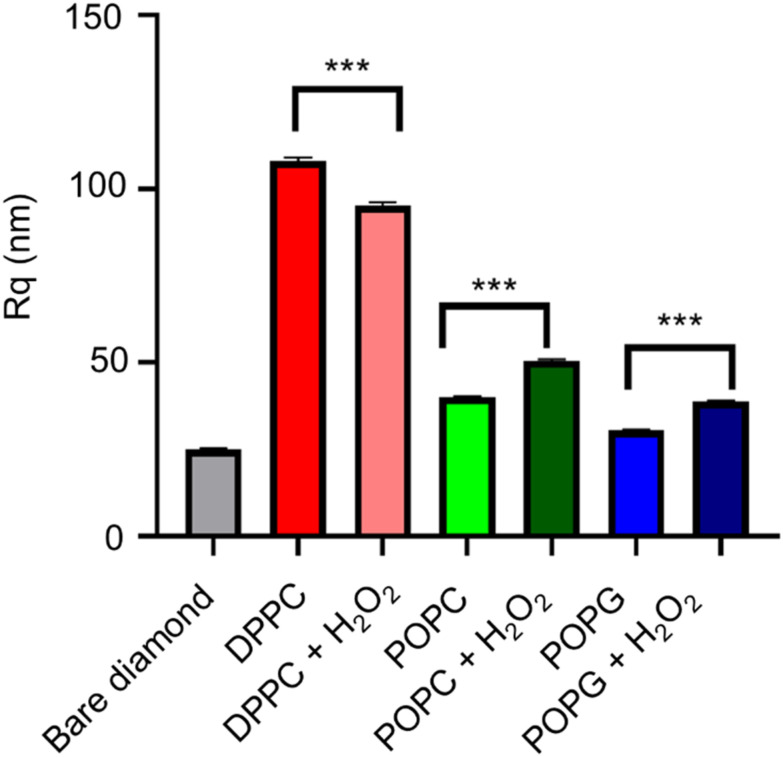
Analysis of the roughness of the AFM images. Statistical significance (*N* = 5) was assessed with an unpaired *t*-test, having found statistical significance with all pairs. The bare diamond sample has a *p* < 0.001 (****) significant difference with all the pairs.

The AFM images from the samples before and after their exposure to the oxidizing agent (Fig. 9), alongside their mean roughness (measured by the *R*_q_ parameter, Fig. 10).

Although all of the samples show a significant change in the mean roughness, only the membranes with the double bonds show an increase in roughness, which is consistent with the same measurements performed in similar peroxidised lipid bilayers.^28^

### 3.6. XPS spectrum of the samples

**Fig. 11:**
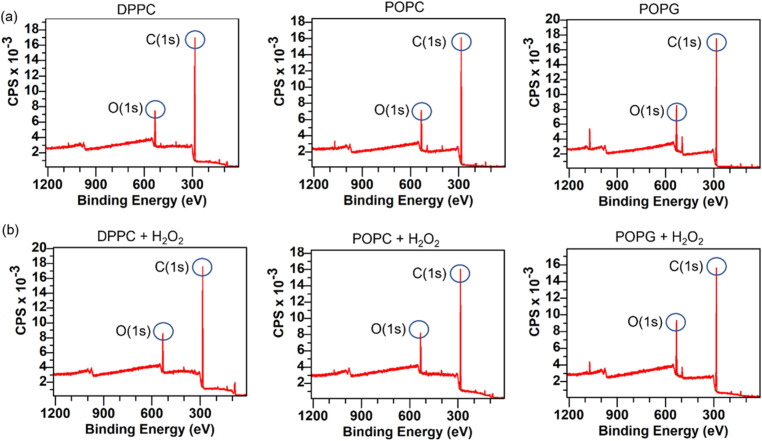
XPS spectra for the membranes before and after H_2_O_2_. The row (a) on top shows the spectra for the samples before being exposed to the oxidizing agent, while the row (b) on bottom displays the spectra from the same sample after being exposed to H_2_O_2_.

**Fig. 12:**
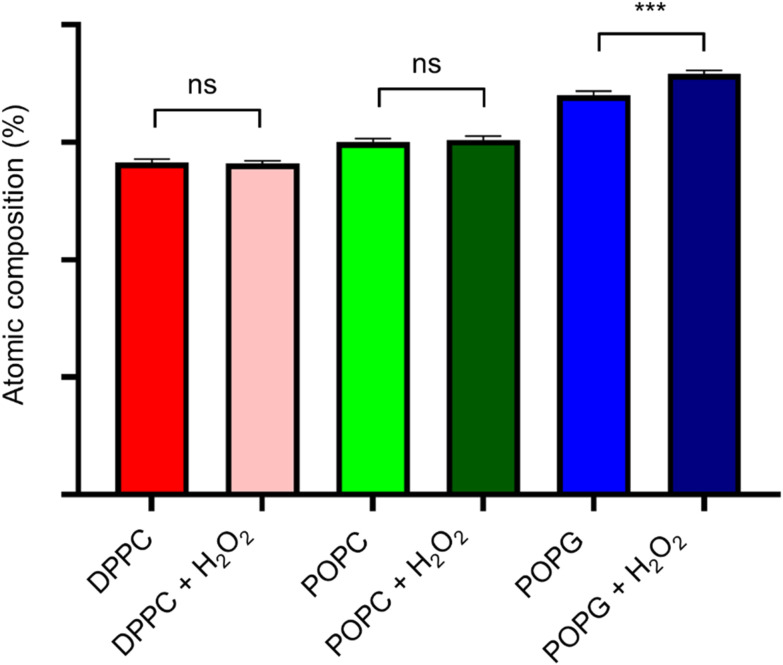
Atomic composition difference for all the membranes in the oxygen peak. Statistical significance (*N* = 10) was assessed with an unpaired *t*-test, having statistical significance only for the POPG pair.

XPS spectra of the lipid bilayers before and after being exposed to H_2_O_2_ (2% v/v) are shown in Fig. 11. As expected, the main peaks are present in the C(1s) region, while the next peak is in the O(1s) region. In order to retrieve information about the lipid peroxidation process, we proceed to compare the atomic composition and evaluate their changes in the O(1s) peak (Fig. 12).

The surface spectroscopy of the membranes shows an increase in the quantity of oxidized groups only for the POPG lipid, but not for the other groups. While it is expected that no significant change is present in the DPPC lipid, the XPS spectrum is unable to detect the lipid peroxidation in the POPC membrane. This might be due to oxygen from the air adsorbing to the surface or simply due to the fact that we have a very thin submonolayer.

## 4. Conclusions

We have studied the physical and chemical properties of lipid bilayers supported on a diamond sensor, including a method that is new for detecting lipid peroxidation. We have shown that relaxometry is a powerful tool to measure lipid peroxidation. From bulk diamond experiments where we observed a 250 × 250 micrometer lipid bilayer we are able to determine some information on the nature of the lipid bilayer and observe to what extent the lipids are peroxidised. Further, it is possible to perform such measurements with a nanoscale spatial resolution by analysing the data pixel by pixel, in such a way that we are able to visualise and differentiate between lipid rafts with different properties.

It is important to note that the method in the current form cannot provide spectroscopic information, however it is possible to recover such type of information by integrating cross-relaxometry to the measurement protocol.^29,30^ Another important aspect of this study is in how to use this method in order to sense lipid peroxidation in a real, cellular membrane, it is important to take into consideration the behaviour of other organelles in the cell, but also how the surface of the diamond sensor will interact with the medium.^31^

## Conflicts of interest

There are no conflicts to declare.

## Supplementary Material

NR-015-D3NR01167D-s001
