# Supplementary material for: Lipid peroxidation in diamond supported bilayers
Source: Nanoscale. 2023 Apr 11;15(17):7920–8. doi: 10.1039/d3nr01167d (PMC10273028; doi:10.1039/d3nr01167d)
Supplement: NR-015-D3NR01167D-s001 [file NR-015-D3NR01167D-s001.pdf]

Electronic Supplementary Material (ESI) for Nanoscale.
This journal is © The Royal Society of Chemistry 2023

Supplementary Information:

Lipid peroxidation in diamond supported bilayers

A. R. Ortiz Moreno[1]#, R.Li[1]#,K. Wu, R. Schirhagl*[1]

1. Groningen University, University Medical Center Groningen, Antonius Deusinglaan 1, 9713 AW Groningen, the Netherlands

E-mail corresponding author:

romana.schirhagl@gmail.com

# these authors contributed equally

1. Relaxometry curves

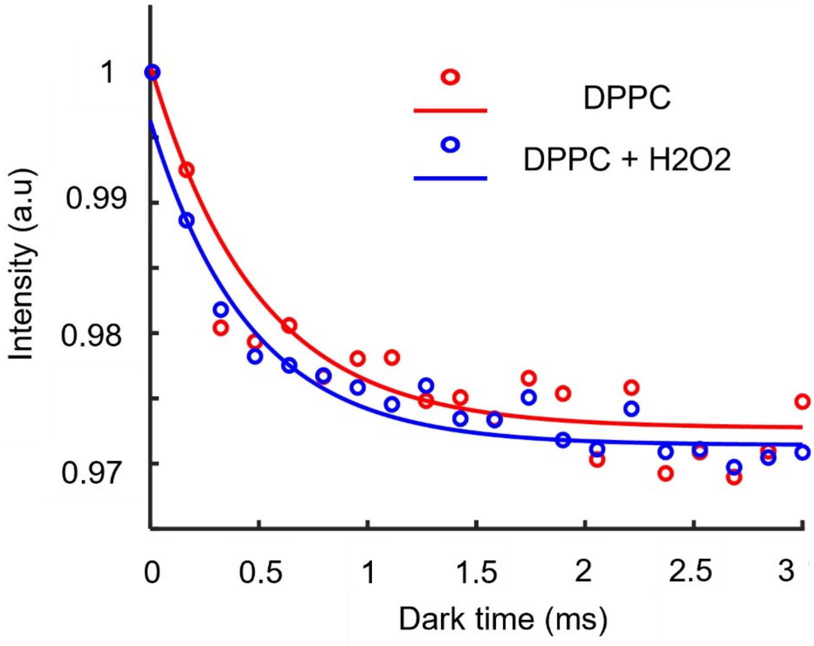

Figure S1: Relaxometry curves for the DPPC measurements (Data in dots, exponential fit in the continuous line).

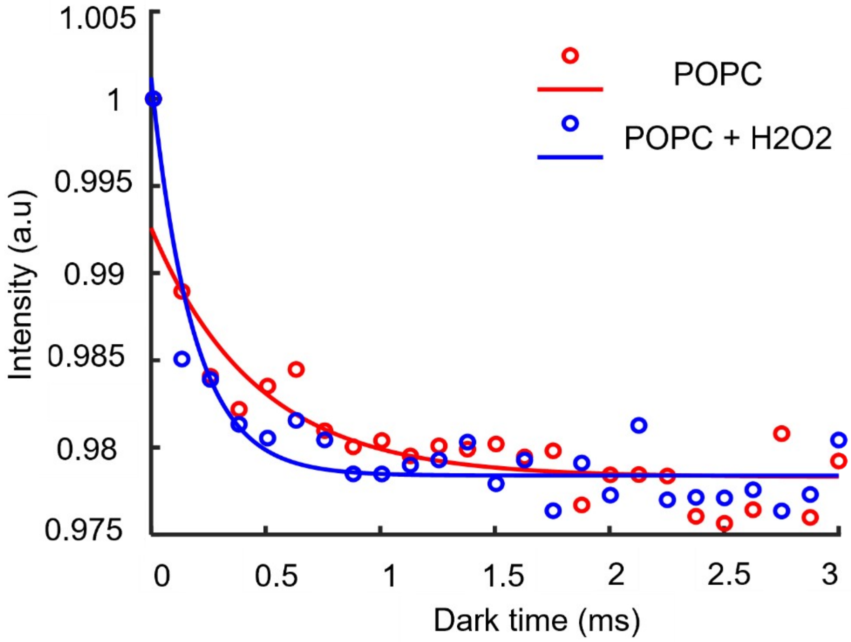

Figure S2: Relaxometry curves for the POPC measurements (Data in dots, exponential fit in the continuous line).

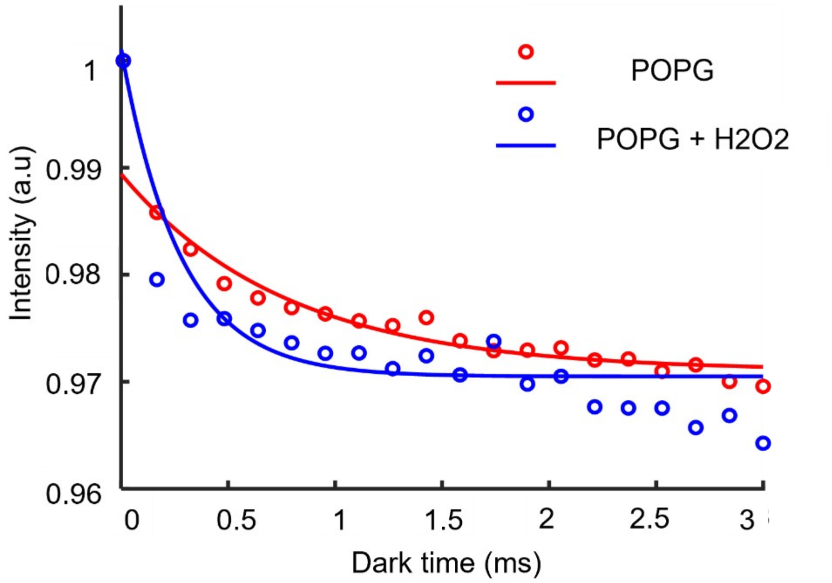

Figure S3: Relaxometry curves for the POPG measurements (Data in dots, exponential fit in the continuous line).

2. Particle size

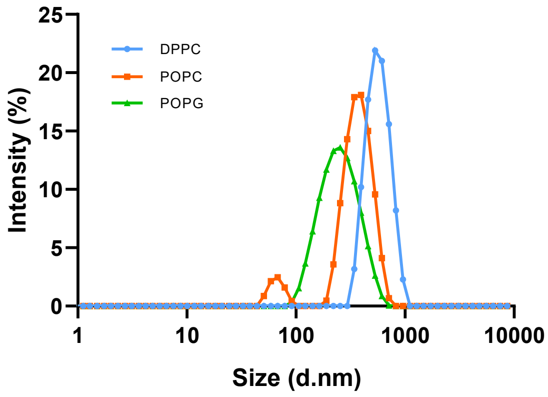

Figure S4. Size distribution of lipid particles measured by DLS

	Z-average /nm	PDI
DPPC	607.8	0.248
POPC	371.8	0.400
POPG	223.7	0.214

3. Surface coverage for the different membrane types

Surface coverage: Fluorescently labelled liposomes where imaged using confocal microscopy (see main manuscript). The image analysis was done using FIJI. 4 areas of 40*40 um^2 were selected randomly. A threshold of 124 was selected to differentiate liposomes from uncovered area.

Percentage of Surface coverage= covered area of liposomes/ 40*40 um^2 100%

Statistics: using T-test of Mann-Whitney for two groups by GraphPad Prism 8.0.1

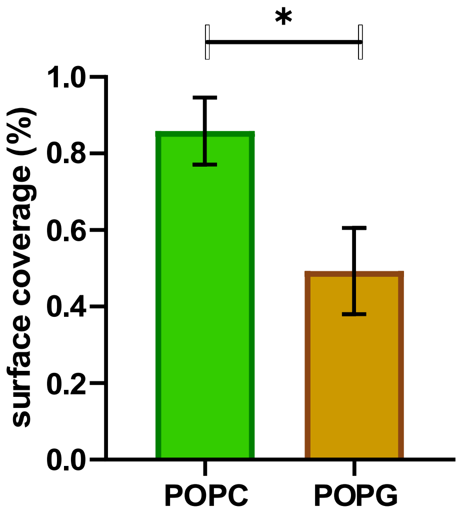

Figure S5. Surface coverage for different lipid membranes of the diamond surface. (n=4)
